# New estimates of the storage permanence and ocean co-benefits of enhanced rock weathering

**DOI:** 10.1093/pnasnexus/pgad059

**Published:** 2023-04-04

**Authors:** Yoshiki Kanzaki, Noah J Planavsky, Christopher T Reinhard

**Affiliations:** School of Earth and Atmospheric Sciences, Georgia Institute of Technology, Atlanta, GA 30318, USA; Department of Earth and Planetary Sciences, Yale University, New Haven, CT 06511, USA; School of Earth and Atmospheric Sciences, Georgia Institute of Technology, Atlanta, GA 30318, USA

**Keywords:** carbon cycle, climate change, negative emissions technology

## Abstract

Avoiding many of the most severe consequences of anthropogenic climate change in the coming century will very likely require the development of “negative emissions technologies”—practices that lead to net carbon dioxide removal (CDR) from Earth's atmosphere. However, feedbacks within the carbon cycle place intrinsic limits on the long-term impact of CDR on atmospheric CO_2_ that are likely to vary across CDR technologies in ways that are poorly constrained. Here, we use an ensemble of Earth system models to provide new insights into the efficiency of CDR through enhanced rock weathering (ERW) by explicitly quantifying long-term storage of carbon in the ocean during ERW relative to an equivalent modulated emissions scenario. We find that although the backflux of CO_2_ to the atmosphere in the face of CDR is in all cases significant and time-varying, even for direct removal and underground storage, the leakage of initially captured carbon associated with ERW is well below that currently assumed. In addition, net alkalinity addition to the surface ocean from ERW leads to significant increases in seawater carbonate mineral saturation state relative to an equivalent emissions trajectory, a co-benefit for calcifying marine organisms. These results suggest that potential carbon leakage from the oceans during ERW is a small component of the overall ERW life cycle and that it can be rigorously quantified and incorporated into technoeconomic assessments of ERW at scale.

**Significance**

Avoiding the most severe consequences of anthropogenic climate change in the coming century will likely require “negative emissions technologies”—practices leading to removal of carbon dioxide from Earth's atmosphere. Ideally, these approaches will be scalable, traceable, and permanent. Here we explicitly quantify the permanence of CO_2_ removal from the atmosphere during enhanced rock weathering (ERW) with a large ensemble of Earth system models. We find that the vast majority of CO_2_ captured through ERW is stored permanently within the ocean interior, and that long-term capture efficiency can be robustly incorporated into economic models for fostering CO_2_ removal at scale.

## Introduction

Realistic pathways to a maximum of 1.5 °C or 2 °C of mean global warming since the start of the industrial period will require both a rapid transition to net zero greenhouse gas emissions and sustained carbon dioxide removal (CDR) from the atmosphere (1, 2). The extent of CDR required to meet a given climate goal will depend on how quickly and aggressively anthropogenic greenhouse gas emissions can be reduced, but there is increasingly high confidence across scenarios that time-averaged rates of CDR on the order of ∼5–10 gigatons (Gt = 10^9^ tons) of CO_2_ per year will be required through the end of the century to meet climate goals (2). These considerations have motivated considerable discussion of negative emissions technologies (NETs)—approaches that remove and sequester CO_2_ from Earth's atmosphere (3). However, deployment of CDR at scale—whether driven primarily by voluntary markets (4) or by a broader policy mix (5–7)—will require robust and accurate understanding of removal permanence and transparent mechanisms for removal accounting.

NETs can be expected to vary significantly with respect to long-term capture permanence. Solubility trapping of atmospheric CO_2_ as dissolved inorganic carbon (DIC) (principally bicarbonate, ${\mathrm{HCO}}_{3}^{-}$) can be catalyzed through enhanced rock weathering (ERW), either in terrestrial or marine systems. One of the main potential advantages of this process is that the ${\mathrm{HCO}}_{3}^{-}$ produced during the weathering reaction can be stored in the oceans on timescales approaching that of marine carbonate compensation (e.g. ∼10^4^ years; (8–10)). In principle, this stability makes enhanced weathering distinct from many other forms of natural carbon sequestration—such as afforestation/reforestation and enhanced soil carbon storage—for which biomass burning (11), shifts in management practice (12, 13), or changes in soil respiration rates (14) can lead to significant, rapid, and often unpredictable release of CO_2_ back to the atmosphere. However, the ${\mathrm{HCO}}_{3}^{-}$ entering the oceans from ERW will engage with the seawater carbonic acid system and some fraction of this initially captured carbon could be returned to the atmosphere. This carbon re-emission is distinct from the well-known Earth system response to CDR in which carbon stored by the oceans and the terrestrial biosphere “leaks” back into the atmosphere, effectively undoing some fraction of initially deployed CDR (15, 16). The overall fraction of carbon released back to the atmosphere and the release timescales of this process are poorly known.

Here, we assess the long-term storage permanence of carbon captured through ERW, explicitly distinguishing this carbon flow from the natural relaxation of Earth's carbon cycle in response to either significant emissions reductions or negative CO_2_ emissions. Specifically, we use a large ensemble of Earth system model simulations to compare the long-term backflux of legacy anthropogenic carbon in response to emissions reduction or direct CO_2_ removal with that of an equivalent mitigation through ERW. This allows us to distinguish between the baseline carbon cycle response to negative/mitigated emissions and the re-release of previously captured carbon through ERW. We find that the long-term storage of captured carbon as DIC in the ocean interior is ∼90% efficient, and that leakage of initially captured through ERW back to the atmospheric CO_2_ pool on decadal timescales is well-constrained and is significantly below that currently assumed. This work also provides new estimates of the extent to which solubility trapping of CO_2_ as ${\mathrm{HCO}}_{3}^{-}$ during ERW results in mitigation of surface ocean acidification relative to the same level of direct CDR or mitigated emissions.

## Methods

**Fig. 1.**
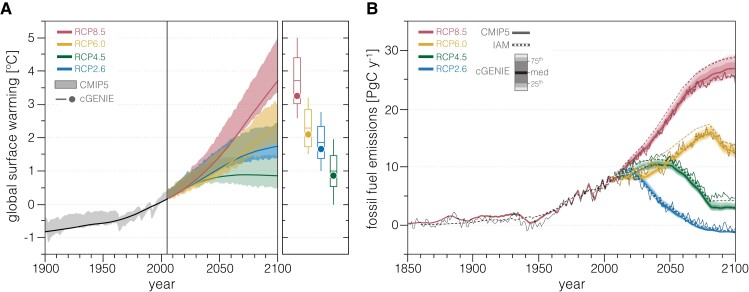
Historical and future trajectories for temperature (left) and fossil fuel emissions (right) from an Earth system model of intermediate complexity (EMIC) compared to results from the CMIP5. Solid lines at left show cGENIE temperature trajectories for each RCP scenario, while shaded regions show corresponding ranges for CMIP5 models (22). Box plots (ensemble mean, ± 1 SD, and minimum to maximum range) show CMIP5 results for globally averaged temperature between 2080 and 2099 (22), while filled circles show cGENIE results. Solid lines and error envelopes at right show inverted fossil fuel CO_2_ emissions for each RCP scenario from the cGENIE ensemble compared with ensemble mean trajectories from CMIP5 and a series of IAMs (23).

We explore the impacts of large-scale CDR using a “carbon-centric” version of the Grid Enabled Integrated Earth system model—cGENIE. The ocean physics and climate model components of cGENIE comprise a reduced physics (frictional geostrophic) 3D ocean circulation model coupled to a 2D energy-moisture balance model (EMBM) and a dynamic-thermodynamic sea ice model. The ocean biogeochemistry module includes a sophisticated ocean carbon cycle including a fully coupled carbonate system with shallow sediment diagenesis, which allows the ocean inorganic carbon cycle to be run as an open system with delivery from weathering of the land surface and ultimate burial as calcite (CaCO_3_) in marine sediments. Full descriptions of the climate model and ocean physics can be found in Refs. (17, 18), while more detailed description and validation of the ocean and sediment biogeochemistry is provided in Refs. (19–21). The climate model response in cGENIE for control runs of the benchmark representative concentration pathway (RCP) is consistent with Coupled Model Intercomparison Project Phase 5 (CMIP5) projections to year 2100 (Fig. 1A), while gridded fields of the principal carbonate system variables, including dissolved concentrations of DIC and alkalinity, pH, aragonite saturation state, also compare well with historical observations of surface ocean chemistry (Fig. S1).

The response of the terrestrial biosphere and soil carbon reservoir to different climate and emissions scenarios varies across existing CMIP-class models. To explore the effect of this process on the efficiency of enhanced weathering, we implement a simple model of carbon exchange with the terrestrial biosphere in which aboveground biomass (vegetation) and soil carbon are treated as global pools that respond to temperature and atmospheric *p*CO_2_ (e.g. a “slab” or “box” terrestrial biosphere, similar in design to that of Ref. (24)). The model tracks changes in the size of the aboveground carbon reservoir (vegetation) and soil carbon, with vegetation storage driven primarily by atmospheric *p*CO_2_ and soil carbon storage driven primarily by temperature (see Methods; Table S1). We stochastically vary the parameters of our slab biosphere across the ranges given in Table S2, then subsample parameter sets that yield results that are consistent with modern observations of soil carbon stocks, aboveground net primary production, and vegetation turnover time and result in a dynamic response that falls within the range of CMIP5 projections for changes in vegetation and soil organic carbon pools over the coming century (see Supplementary Information). The slab biosphere model is then driven by global average land temperature (excluding Antarctica and Greenland) and atmospheric *p*CO_2_ values derived from the cGENIE model. This process yields our final large ensemble of *n* = 980 Earth system model simulations.

The model climate system and ocean carbonate/alkalinity cycle are spun up to steady state using a two-stage procedure. First, the model is run as a closed system for 20 ky with atmospheric abundances of CO_2_, CH_4_, and N_2_O imposed at preindustrial values to bring the ocean-atmosphere system and shallow sediments into steady state. This run is used to diagnose the approximate steady state burial flux of calcium carbonate in marine sediments, which is then imposed as a weathering flux of calcium and alkalinity in a second stage spinup in which the ocean and sediments are allowed to evolve as an open system. The second stage spinup is run for 20 ky to allow the long-term ocean alkalinity budget to achieve steady state. All subsequent simulations are branched (restarted) from the open system spinup at model year 1765 and run to year 2300 according to the RCP and extended concentration pathway (ECP) scenarios for atmospheric CO_2_, CH_4_, and N_2_O (25). Time-varying atmospheric abundances of CH_4_ and N_2_O are imposed according to a given RCP/ECP trajectory for all simulations, while atmospheric CO_2_ abundance is emission-driven. The emission trajectory for a given RCP is first computed by the model by prescribing the atmospheric CO_2_ trajectory for that scenario, with all subsequent runs utilizing the emission trajectory diagnosed for each RCP/ECP pathway. The historical and future fossil fuel emissions diagnosed by control simulations in our large ensemble compare well with CMIP5 ensemble mean values and integrated assessment model (IAM) results (Fig. 1B).

We compare two end-member styles of carbon cycle intervention, designed to pinpoint the carbon fluxes associated with CDR through ERW and to explicitly deconvolve these from the well-known return fluxes of carbon from the ocean and terrestrial biosphere back into the atmospheric CO_2_ pool that result naturally from negative (or mitigated) CO_2_ emissions. In the first, we instantaneously reduce CO_2_ emission rates by a specified value (in GtCO_2_ y^−1^) relative to the control emissions for a given RCP (Fig. 1B). These simulations are referred to here as “baseline” (base) simulations. Mechanistically, these could represent either direct mitigation of anthropogenic emissions beyond those implied by a given RCP trajectory or the permanent removal of CO_2_ from the atmosphere, for example through direct air capture and underground storage (26). In the second, we specify an initial CDR rate (in GtCO_2_ y^−1^) which is then translated into a removal of CO_2_ from the atmosphere and a corresponding river-routed flux of DIC and alkalinity to the coastal ocean. The key difference between these styles of intervention for our purposes is that in the ERW case, carbon is transiently repartitioned into a non-radiative but potentially labile form of surface carbon which can degas back to the atmosphere across a range of timescales from decades to millennia (20, 27).

We simulate ERW using two distinct feedstocks with idealized stoichiometries for natural silicate and carbonate minerals (28):


(1) $$\mathrm{CaSi}O_{3}+2CO_{2}+3H_{2}O\to Ca^{2\;+\;}+{2\mathrm{HCO}}_{3}^{-}+H_{4}\mathrm{Si}O_{4},$$

(2) $$\mathrm{CaC}O_{3}+CO_{2}+H_{2}O\to Ca^{2\;+\;}+{2\mathrm{HCO}}_{3}^{-}.$$

Fluxes of DIC and alkalinity to the ocean per ton of CO_2_ removed for enhanced silicate weathering (ESW) and enhanced carbonate weathering are controlled according to the stoichiometries shown in Eqs. 1 and 2, respectively. Note that on very long timescales (>10^5^ years), the formation and burial of carbonate minerals in marine sediments (e.g. the reverse of Eq. 2) releases CO_2_, undoing most or all of the capture associated with ERW using carbonate rock and around half of the capture associated with ERW using silicate feedstock. Although evaluating the likelihood of surmounting technological and/or institutional barriers to scale is beyond the scope of the current work, we examine the response of the Earth system to a range of CDR deployment levels by implementing a range of initial CDR rates between 0.5 and 40 GtCO_2_ y^−1^. This is meant to be broadly inclusive of relatively modest CDR deployment to a deployment scale sufficient to offset a large fraction of global anthropogenic CO_2_ emissions (currently ∼40 GtCO_2_ y^−1^ including emissions due to changes in land use) (3, 29–31).

We quantify the net impact of carbon cycle intervention *i* on atmospheric CO_2_ (*η_i_*) by comparing the time-integrated flux due to CO_2_ removal (or mitigated emissions) with the time-integrated release of carbon from the ocean and terrestrial biosphere relative to a corresponding control simulation lacking carbon cycle intervention:


(3) $$\eta_{i}=\int_{2030}^{\;t}\left[J_{\mathrm{CDR},i}-(J_{\mathrm{sea}\text{-}\mathrm{air}}^{\exp,i}-J_{\mathrm{sea}\text{-}\mathrm{air}}^{\mathrm{ctrl}})-(J_{\mathrm{lnd}\text{-}\mathrm{air}}^{\exp,i}-J_{\mathrm{lnd}\text{-}\mathrm{air}}^{\mathrm{ctrl}})\right]dt^{\prime }/\int_{2030}^{\;t}J_{\mathrm{CDR},i}\;dt^{\prime },$$

where *J*_CDR_ is the assumed CDR deployment level (or additional mitigated emissions relative to a given RCP scenario; GtCO_2_ y^−1^), *J*_sea-air_ is the global CO_2_ outgassing flux from the ocean to the atmosphere (GtCO_2_ y^−1^), *J*_lnd-air_ is the global CO_2_ flux from the terrestrial biosphere and soil carbon pool, *t* is time, and the superscripts “exp” and “ctrl” refer to simulations with and without CDR deployment (or additional mitigated emissions), respectively. This is conceptually similar to the “perturbation airborne fraction” of (16) and represents the time-integrated response of ocean and terrestrial carbon reservoirs to negative (or mitigated) emissions. We then estimate the carbon backflux for each scenario *i* (*p_i_*), expressed here as a percentage relative to the deployed CDR or mitigated emissions level:


(4) $$p_{i}=[1-\eta_{i}]\cdot 100\text{\%}.$$

Lastly, we define a “leakage” term for ERW scenarios that is normalized to that for an equivalent level of CDR or mitigated emissions:


(5) $$p_{\mathrm{leak}}=p_{\mathrm{ERW}}-p_{\mathrm{base}}.$$

Mechanistically, this represents an additional return flow of carbon into the atmosphere during ERW scenarios beyond that associated with the baseline Earth system readjustment to direct CO_2_ removal or mitigated emissions. It thus explicitly diagnoses carbon that is initially captured through ERW but then re-released to the atmospheric CO_2_ pool.

## Results and discussion

**Fig. 2.**
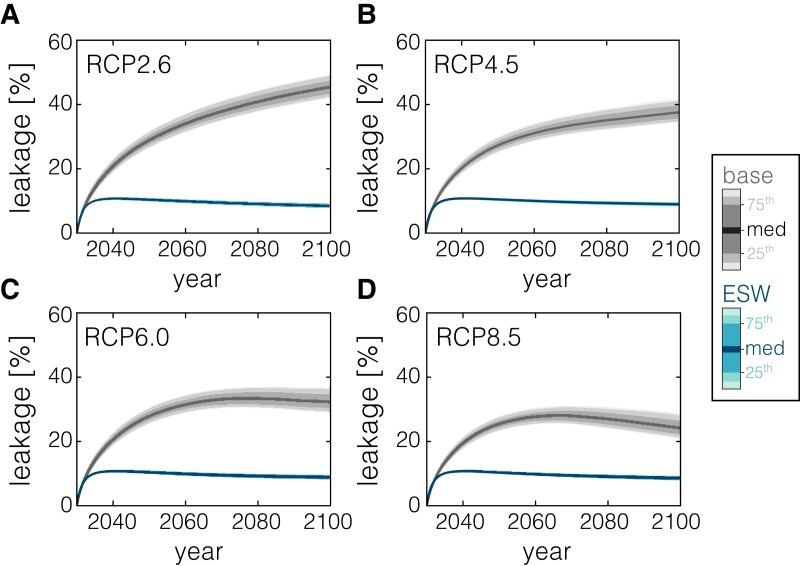
Carbon backflux through 2100 across a range of emission scenarios, shown as a time-integrated percentage relative to CDR deployment level. Curves and error envelopes show the ensemble median and uncertainty on baseline (modulated emissions) response and carbon leakage during ESW after correcting to the baseline response. Values for all scenarios are shown for a CDR deployment level of 10 GtCO_2_ y^−1^, though the relative magnitude of leakage is only weakly sensitive to CDR deployment level (see Supplementary Information).

Baseline Earth system carbon backflux and residual ERW carbon loss over time for a CDR deployment/mitigation level of 10 GtCO_2_ y^−1^ are shown in Fig. 2 for scenarios simulating ESW. The baseline Earth system adjustment includes significant release of carbon from the ocean and terrestrial/soil carbon reservoirs in response to negative or mitigated CO_2_ emissions, which effectively counteracts a significant fraction of initial CO_2_ capture or additional mitigated emissions. For example, 45 ± 4% of initially captured/mitigated CO_2_ is counteracted by CO_2_ backflux from natural carbon sinks in a low-emissions scenario at the end of the century (Fig. 2A). This drops to 24 ± 4% for a high-emissions scenario (Fig. 2D) because of enhanced degassing of legacy anthropogenic carbon from the surface ocean and terrestrial/soil pools in scenarios with stronger mitigation, consistent with previous Earth system modeling of the carbon cycle response to negative emissions (15, 16, 32, 33). Importantly, we find that the magnitude of relative carbon backflux during CDR deployment is largely rate-independent across nearly two orders of magnitude (Figs. S7 and S8).

The additional release of carbon in ERW scenarios relative to that occurring during an equivalent magnitude of direct removal/storage or mitigated emissions stabilizes rapidly (Fig. 2) and is relatively invariant across the emissions pathways examined here. This additional inefficiency in capture amounts to ∼9 ± 1% for deployment of silicate feedstock and around twice this for carbonate feedstock (Fig. 2A–D and Fig. S8). This carbon leakage is also insensitive to the overall implemented capture rate (see Figs. S7 and S8). Carbon leakage increases to 19 ± 1% when using carbonate rock as a feedstock for ERW (see Eq. S22 and Fig. S7). These results indicate that the vast majority of CDR inefficiency in ERW scenarios is driven by the release of carbon from the surface ocean and terrestrial/soil pools rather than net repartitioning of previously captured carbon back into the atmosphere.

**Fig. 3.**
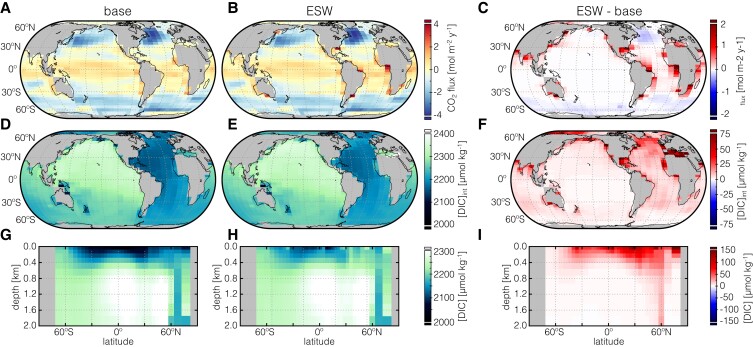
Ocean carbon cycle response to CDR in an EMIC. Shown at left are sea-air CO_2_ fluxes (top), depth-integrated inventory of dissolved inorganic carbon ([DIC]_int_; middle), and zonally averaged DIC concentrations (bottom) for the baseline (modulated emissions) case and the ESW scenario. Shown at right are anomaly plots of sea-air flux (top), depth-integrated DIC inventory (middle), and zonally averaged DIC (bottom) between the ESW scenario and the equivalent modulated emissions case. Results are shown for year 2070 of an RCP4.5 emissions trajectory and a continuous CDR deployment level of 10 GtCO_2_ y^−1^ starting in 2030 (see Supplementary Information). Note that DIC concentration/anomaly results (D–I) are shown excluding the uppermost grid cell (80 m).

We observe clear increases in sea-air CO_2_ flux in ESW simulations relative to an equivalent baseline CDR/mitigation case, with increases in CO_2_ outgassing focused near river mouths where inputs of DIC and alkalinity are concentrated (Fig. 3B and C). However, we observe clear signals for effective carbon storage in the ocean interior in ERW simulations, including significant column-integrated DIC storage below the mixed layer (Fig. 3D–F) and strong anomalies in DIC concentration that penetrate to significant ocean depths on decadal timescales (Fig. 3G–I). The overall result is that ∼90% of the carbon initially captured through ESW remains stored as DIC in the ocean on 10^2^–10^3^-year timescales (Fig. 2). This is a significant shift in the magnitude and uncertainty of ocean carbon leakage associated with ERW and is largely insensitive to emissions pathway (Figs. S9–S11). For instance, previous estimates based on thermodynamic considerations suggested that up to 30% of the initial carbon captured during this process could be lost during marine CO_2_ evasion (34).

**Fig. 4.**
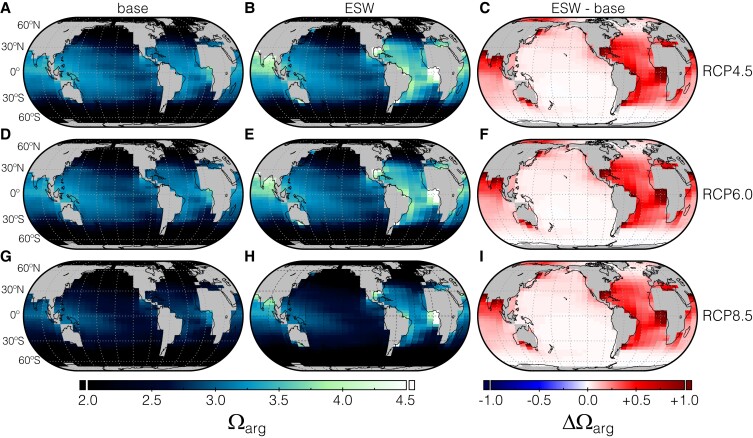
The response of surface ocean aragonite saturation state (Ω_arg_) to CDR in an EMIC. Shown at left are surface Ω_arg_ values for the modulated emission (base) and ESW scenarios for RCP4.5 (top), RCP6.0 (middle), and RCP8.5 (bottom). Shown at right are anomaly plots of Ω_arg_ between the ESW scenario and the equivalent modulated emissions case. Results are shown for year 2070 of a given emissions trajectory and a continuous CDR deployment level of 10 GtCO_2_ y^−1^ starting in 2030.

ERW exerts significantly more leverage on global and regional surface ocean carbonate chemistry than an equivalent level of direct CO_2_ removal or mitigated emissions (Fig. 4). For instance, ESW leads to a ∼20% increase in global average surface ocean aragonite saturation state relative to the control case, which is more than double the impact of direct CO_2_ removal or mitigated emissions (Figs. S6 and S12). Regional differences are also significant (Fig. 4), with large regions of the surface ocean maintaining aragonite saturation states above ∼2.5 even in the high-emission scenario if CDR is accomplished through ESW. These patterns are accentuated when using carbonate feedstock for ERW (Figs. S6 and S12). Regional increases in aragonite saturation state in ERW cases relative to direct removal or mitigated emissions are often very large (Fig. 4C, F, and I) and are only weakly sensitive to emissions trajectory and removal/mitigation level. The threshold saturation state below which coral reef systems become non-viable is uncertain (35, 36) and surface coral reef systems face sobering challenges connected to warming temperatures that arguably no NET is equipped to address (37). Nevertheless, extensive (>10 GtCO_2_ y^−1^) deployment of ERW has the potential to maintain aragonite saturation states above values that would be expected to maintain active coral calcification (38–40) even in moderate- and high-emission scenarios (Fig. 4).

## Conclusions and future directions

Our results add further support to the prediction that release of carbon from the ocean and terrestrial reservoirs will act to counteract a significant fraction of negative or mitigated CO_2_ emissions in the coming century. In this sense, the main outcome of efforts directed at deploying CDR at scale should be thought of as sequestering carbon from the combined atmosphere-ocean-biosphere system rather than as directly and permanently removing CO_2_ from the atmosphere. Although this distinction may be implicitly accounted for in Earth system and IAM estimates of the levels of CDR required to achieve a given temperature threshold (2), it is very often not explicitly made. Given potential differences in the representation of carbon cycle processes across models (and thus the potential for differing Earth system response to negative or mitigated emissions), we suggest that analyses and forecasts of the impacts of CO_2_ removal (and mitigated emissions) should explicitly delineate between gross and net carbon cycle impacts. This would promote the development of more precise estimates of the implied technoeconomics of different CDR strategies, aid in diagnosing differences in carbon cycle response between models, and streamline communication within and between stakeholder communities.

Our results also suggest that the long-term ocean storage of carbon as DIC during ERW is well-constrained, efficient, and largely insensitive to emissions trajectory or CDR deployment level. This result contrasts with the currently accepted view that 15–30% of the carbon initially captured through ERW will flow back into the atmosphere on decadal timescales (30, 34, 41–43). We suggest that this difference arises straightforwardly from previous estimates treating the surface ocean as a single reservoir that is uniformly at thermodynamic and gas exchange equilibrium with the atmosphere. In contrast, allowing for three-dimensional transport of DIC and regionally variable departures from gas exchange equilibrium results in more effective injection and storage of captured carbon in the ocean interior. Exploring this process with higher-resolution, fully coupled ocean biogeochemistry models will be an important avenue for future work. In addition, developing better constraints on the potential for carbon leakage during transport from deployment locations to the ocean is a critical topic for future research (44, 45). Nevertheless, our results indicate that the overall loss of initially captured carbon from the ocean is a small fraction of the ERW life cycle and that it is potentially predictable to a relatively high degree of precision. In principle, this renders it tractable to robustly discount dollar-per-ton costs of CO_2_ removal through ERW for long-term ocean storage efficiency.

Lastly, our results provide impetus to consider cost incentives for mitigation of anthropogenic carbon in the surface ocean as distinct from capture and removal of CO_2_ from the atmosphere. Earth's oceans have taken up ∼30% of anthropogenic fossil fuel emissions since the industrial age (46, 47), and this uptake continues to drive ocean acidification (48). The future impacts of this will potentially devastate marine ecosystems that provide havens for biodiversity (49) and a wide range of other economic benefits and ecosystem services (50, 51). Development of an “ocean alkalinity market” that is not directly used for annual offsets or other comparable incentives directed specifically at mitigating ocean acidification may provide a more effective way of accurately valuing different NETs with contrasting impacts on surface ocean chemistry. In any case, our results suggest that different CDR approaches can have very different impacts on the surface ocean alkalinity budget and carbonate mineral saturation states for comparable amounts of CO_2_ removal from the atmosphere, which should be taken into consideration when evaluating the overall impacts and cost-benefit profile of varying CDR strategies.

## Materials and methods

### Earth system model

We explore the impacts of large-scale CDR using a “carbon-centric” version of the Grid ENabled Integrated Earth system model—cGENIE. The ocean physics and climate model components of cGENIE comprise a reduced physics (frictional geostrophic) 3D ocean circulation model coupled to a 2D EMBM and a dynamic-thermodynamic sea ice model (18). Heat, salinity, and biogeochemical tracers are transported via parameterized isoneutral diffusion and eddy-induced advection (17). The ocean model exchanges heat and moisture with the atmosphere, sea ice, and land while being forced at the ocean surface by zonal and meridional wind stress according to a specified static wind field. Heat and moisture are horizontally mixed throughout the atmosphere and exchange heat and moisture with the ocean and land surfaces, with precipitation occurring above a given relative humidity threshold. The sea ice model tracks horizontal ice transport and exchanges of heat and fresh water, using the thickness, areal fraction, and concentration of ice as prognostic variables. Full descriptions of the climate model and ocean physics can be found in Refs. (17, 18). The ocean model is configured here as a 36 × 36 equal-area grid (uniform in longitude and sine of latitude) with 16 logarithmically spaced depth levels and seasonal forcing at the ocean surface.

The ocean and sediment biogeochemistry modules in cGENIE control air-sea gas exchange, the transformation and repartitioning of biogeochemical tracers within the ocean, and the impacts of shallow sediment diagenesis on calcium carbonate formation/dissolution and burial. The ocean biological carbon pump is driven by a parameterized uptake rate of nutrients in the surface ocean, with this flux converted stoichiometrically to biomass that is then partitioned into particulate or dissolved organic matter for downstream advective transport, sinking, and remineralization within the ocean interior. Dissolved organic matter is transported with the ocean circulation and decays according to a specified time constant, while particulate organic matter is instantaneously exported from the surface ocean and is remineralized within the ocean interior following an exponential decay function with a specified remineralization length scale. The ocean biogeochemistry also contains a fully coupled carbonate system, which tracks individual DIC species, dissolved alkalinity, and ocean pH. Calcium carbonate forms in surface ocean grid cells at a stoichiometric ratio with organic matter production (the so-called “rain ratio”) and is exported as a solid species and is dissolved in the ocean interior or shallow marine sediments depending on ambient temperature, pressure, and carbonate chemistry (20, 52). A simple scheme for shallow sediment diagenesis allows us to run the ocean alkalinity cycle as an open system, with delivery from weathering of the land surface and ultimate burial as calcite (CaCO_3_) in marine sediments. More detailed description and validation of the ocean and sediment biogeochemistry in cGENIE is provided in Refs. (19, 21).

### Terrestrial carbon exchange

We implement a simple model of carbon exchange with the terrestrial biosphere in which aboveground biomass (vegetation) and soil carbon are treated as global pools that respond to temperature and atmospheric *p*CO_2_ (e.g. a “slab” or “box” terrestrial biosphere). The model tracks changes in the size of the aboveground carbon reservoir (vegetation, *V*) and soil carbon (*S*) according to


$$\frac{dV(t)}{dt}=N(t)-L(t),$$

$$\frac{dS(t)}{dt}=L(t)-R(t),$$

where *N*(*t*) represents net primary production (GtC y^−1^), *L*(*t*) represents the production rate of litterfall (GtC y^−1^), and *R*(*t*) represents soil respiration (GtC y^−1^). Net primary production is parameterized as a function of atmospheric *p*CO_2_ according to


$$N(t)=N_{0}\left[1+B\ln\left(\frac{C(t)}{C_{0}}\right)\right]\;,$$

where *C*(*t*) is atmospheric *p*CO_2_, *N*_0_ is net primary production at a baseline atmospheric *p*CO_2_ (*C*_0_), and *B* is a growth rate parameter. The rate of litterfall production is given by


$$L(t)=V(t)[\Lambda_{\mathrm{veg}}V(t)+\Lambda_{0}{]}^{-1},$$

where the Λ_veg_ and Λ_0_ terms describe an intrinsic turnover time for vegetation (y). Soil respiration is parameterized as a function of temperature according to


$$R(t)=\;\Gamma S(t)Q_{10}^{\frac{\left(T(t)-T_{0}\right)}{10}},$$

where Γ is the annual soil carbon turnover rate at reference temperature *T*_0_ (y^−1^) and *Q*_10_ represents a parameter describing the factor change in soil respiration rate for a 10 °C change in temperature.

Once *C*_0_ and *T*_0_ are defined, the model contains six parameters (*N*_0_, *B*, Λ_veg_, Λ_0_, Γ, and *Q*_10_). We use a stochastic approach to account for uncertainty in the slab biosphere parameterization. First, we randomly generate 2 × 10^6^ parameter sets from the ranges given in Table S2. These parameter sets are implemented in a stand-alone (offline) version of the slab biosphere model driven by temperature and *p*CO_2_ trajectories from RCP and ECP scenarios (25). These parameter sets are then filtered for those that yield results consistent with modern observations of soil carbon stocks, aboveground net primary production, and vegetation turnover time (Fig. S2) and that result in a dynamic response that falls within the range of CMIP5 projections for changes in vegetation and soil organic carbon pools to the end of the century (Figs. S3–S5), yielding a filtered ensemble of *n* = 7,551 parameter sets. A subset (*n* = 3,000) of these parameter sets are then implemented in a set simulations in which the slab biosphere is fully coupled to cGENIE and are again filtered based on modern observations and end-of-century projections to yield our final ensemble of *n* = 980 Earth system model simulations.

### Model spinup, control simulations, and CDR scenarios

The model climate system and ocean carbonate/alkalinity cycle are spun up to steady state using a two-stage procedure. First, the model is run as a closed system for 20 ky with atmospheric abundances of CO_2_, CH_4_, and N_2_O imposed at preindustrial values to bring the ocean-atmosphere system and shallow sediments into steady state. This run is used to diagnose the approximate steady-state burial flux of calcium carbonate in marine sediments, which is then imposed as a weathering flux of calcium and alkalinity in a second stage spinup in which the ocean and sediments are allowed to evolve as an open system. The second stage spinup is run for 20 ky to allow the ocean alkalinity budget to achieve steady state.

All subsequent simulations are branched from the open system spinup at model year 1765 and run to year 2300 according to the RCP and ECP scenarios for atmospheric CO_2_, CH_4_, and N_2_O (25). Time-varying atmospheric abundances of CH_4_ and N_2_O are imposed according to a given RCP/ECP trajectory for all simulations, while atmospheric CO_2_ abundance is emission-driven. The emission trajectory for a given RCP is first computed by the model by prescribing the atmospheric CO_2_ trajectory for that scenario, with all subsequent runs utilizing the emission trajectory diagnosed in cGENIE for each RCP/ECP pathway.

Our simulations of carbon dioxide capture are designed to represent two distinct CDR deployment modes. In simulations of CDR via ERW, we specify an initial capture rate (in GtCO_2_ y^−1^) which is then translated into a removal of CO_2_ from the atmosphere and a corresponding flux of DIC and alkalinity to the coastal ocean. We simulate ERW using two distinct feedstocks, with idealized stoichiometries for natural silicate and carbonate minerals:


$$\mathrm{CaSi}O_{3}+2CO_{2}+3H_{2}O\to Ca^{2\;+\;}+{2\mathrm{HCO}}_{3}^{-}+H_{4}\mathrm{Si}O_{4},$$

$$\mathrm{CaC}O_{3}+CO_{2}+H_{2}O\to Ca^{2\;+\;}+{2\mathrm{HCO}}_{3}^{-}.$$

Fluxes of DIC and alkalinity to the ocean per ton of CO_2_ removed are controlled according to the stoichiometries shown and are routed to the ocean according to simulated topological network data (53). Note that on arbitrarily long timescales, the formation and burial of carbonate minerals in marine sediments (e.g. the reverse of carbonate dissolution) releases CO_2_, undoing most or all of the capture associated with ERW using carbonate rock and around half of the capture associated with ERW using silicate feedstock.

We also simulate an alternative carbon cycle intervention meant to represent direct CDR or mitigation of emissions in excess of that implied by a given RCP pathway, in which we reduce CO_2_ emission rates globally by a specified value relative to the control emission rates for a given RCP. The key difference between this idealized style of CDR deployment and those associated with ERW is that it is assumed that the carbon is instantaneously and permanently removed from the surface system rather than being transiently repartitioned into a non-radiative but potentially labile form of surface carbon (some of which will ultimately be removed from Earth's surface). This style of intervention could be viewed as consistent with a variety of distinct strategies but is most directly analogized to direct air capture and storage or emissions mitigation. We explore a wide range of CDR deployment scales between 0.5 and 40 GtCO_2_ y^−1^, a range meant to be inclusive of relatively modest CDR deployment on the low end to a deployment scale sufficient to offset most or all of global anthropogenic CO_2_ emissions on the other.

## Supplementary Material

pgad059_Supplementary_Data

## Data Availability

The version of the code used in this paper is tagged as release v1.0.0 and has the DOI https://doi.org/10.5281/zenodo.7737196 (Kanzaki et al., 2023). The code is also hosted on GitHub and can be obtained by cloning https://github.com/ctd-cdr/LEO. Base configuration and forcing files are included in the tagged release, while details of configuration, spinup, and large ensemble simulation are given in the README.txt file in the home directory.
