# Supplementary material for: New estimates of the storage permanence and ocean co-benefits of enhanced rock weathering
Source: PNAS Nexus. 2023 Apr 4;2(4):pgad059. doi: 10.1093/pnasnexus/pgad059 (PMC10122414; doi:10.1093/pnasnexus/pgad059)
Supplement: pgad059_Supplementary_Data [file pgad059_supplementary_data.pdf]

Supplementary Information for

New estimates of the storage permanence and ocean co-benefits of enhanced rock weathering

Yoshiki Kanzaki[a], Noah J. Planavsky[b], Christopher T. Reinhard[a*]

[a]School of Earth and Atmospheric Sciences, Georgia Institute of Technology, Atlanta, GA
[b]Department of Earth and Planetary Sciences, Yale University, New Haven, CT

[*]Correspondence to: Christopher T. Reinhard
Email: chris.reinhard@eas.gatech.edu

This PDF file includes:

Supplementary text
Figures S1 to S12
Tables S1 to S2
SI References

Supplementary Information Text

Earth system model. We explore the impacts of large-scale CDR using a 'carbon-centric' version of the Grid Enabled Integrated Earth system model — cGENIE. The ocean physics and climate model components of cGENIE comprise a reduced physics (frictional geostrophic) 3-D ocean circulation model coupled to a 2-D energy-moisture balance model (EMBM) and a dynamic-thermodynamic sea ice model [1]. Heat, salinity, and biogeochemical tracers are transported via parameterized isoneutral diffusion and eddy-induced advection [2]. The ocean model exchanges heat and moisture with the atmosphere, sea ice, and land while being forced at the ocean surface by zonal and meridional wind stress according to a specified static wind field. Heat and moisture are horizontally mixed throughout the atmosphere and exchange heat and moisture with the ocean and land surfaces, with precipitation occurring above a given relative humidity threshold. The sea ice model tracks horizontal ice transport and exchanges of heat and fresh water, using the thickness, areal fraction, and concentration of ice as prognostic variables. Full descriptions of the climate model and ocean physics can be found in [1, 2]. The ocean model is configured here as a 36 x 36 equal-area grid (uniform in longitude and sine of latitude) with 16 logarithmically spaced depth levels and seasonal forcing at the ocean surface.

The ocean and sediment biogeochemistry modules in cGENIE control air-sea gas exchange, the transformation and repartitioning of biogeochemical tracers within the ocean, and the impacts of shallow sediment diagenesis on calcium carbonate formation/dissolution and burial. The ocean biological carbon pump is driven by a parameterized uptake rate of nutrients in the surface ocean, with this flux converted stoichiometrically to biomass that is then partitioned into particulate or dissolved organic matter for downstream advective transport, sinking, and remineralization within the ocean interior. Dissolved organic matter is transported with the ocean circulation and decays according to a specified time constant, while particulate organic matter is instantaneously exported from the surface ocean and is remineralized within the ocean interior following an exponential decay function with a specified remineralization length scale. The ocean biogeochemistry also contains a fully coupled carbonate system, which tracks individual dissolved inorganic carbon (DIC) species, dissolved alkalinity, and ocean pH. Calcium carbonate forms in surface ocean grid cells at a stoichiometric ratio with organic matter production (the so-called "rain ratio") and is exported as a solid species and is dissolved in the ocean interior or shallow marine sediments depending on ambient temperature, pressure, and carbonate chemistry [3, 4]. A simple scheme for shallow sediment diagenesis allows us to run the ocean alkalinity cycle as an open system, with delivery from weathering of the land surface and ultimate burial as calcite ($CaCO_3$) in marine sediments). More detailed description and validation of the ocean and sediment biogeochemistry in cGENIE is provided in [5, 6].

Terrestrial carbon exchange. We implement a simple model of carbon exchange with the terrestrial biosphere in which aboveground biomass (vegetation) and soil carbon are treated as global pools that respond to temperature and atmospheric pCO$_2$ (e.g., a "slab" or "box" terrestrial biosphere). The model tracks changes in the size of the aboveground carbon reservoir (vegetation, V) and soil carbon (S) according to:

$$\frac{dV(t)}{dt} = N(t) - L(t) \ , \qquad \text{[Eq. S1]}$$

$$\frac{dS(t)}{dt} = L(t) - R(t) \ , \qquad \text{[Eq. S2]}$$

Where $N(t)$ represents net primary production (GtC y^{-1}), $L(t)$ represents the production rate of litterfall (GtC y^{-1}), and $R(t)$ represents soil respiration (GtC y^{-1}). Net primary production is parameterized as a function of atmospheric pCO_2 according to:

$$N(t) = N_0\left[1 + B\ln\left(\frac{C(t)}{C_0}\right)\right] , \qquad \text{[Eq. S3]}$$

Where $C(t)$ is atmospheric pCO_2, N_0 is net primary production at a baseline atmospheric pCO_2 (C_0), and B is a growth rate parameter. The rate of litterfall production is given by:

$$L(t) = V(t)\left[\Lambda_{veg}V(t) + \Lambda_0\right]^{-1} , \qquad \text{[Eq. S4]}$$

Where the Λ_{veg} and Λ_0 terms describe an intrinsic turnover time for vegetation (y). Soil respiration is parameterized as a function of temperature according to:

$$R(t) = \Gamma S(t) Q_{10}^{\frac{T(t)-T_0}{10}} , \qquad \text{[Eq. S5]}$$

Where Γ is the annual soil carbon turnover rate at reference temperature T_0 (y^{-1}) and Q_{10} represents a parameter describing the factor change in soil respiration rate for a 10ºC change in temperature.

Once C_0 and T_0 are defined, the model contains six parameters (N_0, B, Λ_{veg}, Λ_0, Γ, and Q_{10}). We use a stochastic approach to account for uncertainty in the slab biosphere parameterization. First, we randomly generate 2 x 10^6 parameter sets from the ranges given in Table S2. These parameter sets are implemented in a stand-alone (offline) version of the slab biosphere model driven by temperature and pCO_2 trajectories from Representative Concentration Pathway (RCP) and Extended Concentration Pathway (ECP) scenarios [7]. These parameter sets are then filtered for those that yield results consistent with modern observations of soil carbon stocks, aboveground net primary production, and vegetation turnover time (Fig. S2) and that result in a dynamic response that falls within the range of CMIP5 projections for changes in vegetation, soil, and total land organic carbon pools to the end of the century (Fig. S3-S5), yielding a filtered ensemble of n = 7,551 parameter sets. A subset (n = 3,000) of these parameter sets were then implemented in a set simulations in which the slab biosphere is fully coupled to cGENIE and are again filtered based on modern observations and end-of-century projections to yield our final ensemble of n = 980 Earth system model simulations.

Model spinup, control simulations, and CDR scenarios. The model climate system and ocean carbonate/alkalinity cycle are spun up to steady state using a two-stage procedure. First, the model is run as a closed system for 20 kyr with atmospheric abundances of CO_2, CH_4, and N_2O imposed at preindustrial values to bring the ocean-atmosphere system and shallow sediments into steady state. This run is used to diagnose the approximate steady state burial flux of calcium carbonate in marine sediments, which is then imposed as a weathering flux of calcium and alkalinity in a second stage spinup in which the ocean and sediments are allowed to evolve as an open system. The second stage spinup is run for 75 kyr to allow the ocean alkalinity budget to achieve steady state.

All subsequent simulations are branched from the open system spinup at model year 1765 and run to year 2300 according to the Representative Concentration Pathway (RCP) and Extended

Concentration Pathway (ECP) scenarios for atmospheric CO_2, CH_4, and N_2O [7]. Time-varying atmospheric abundances of CH_4 and N_2O are imposed according to a given RCP/ECP trajectory for all simulations, while atmospheric CO_2 abundance is emission-driven. The emission trajectory for a given RCP is first computed by the model by prescribing the atmospheric CO_2 trajectory for that scenario, with all subsequent runs utilizing the emission trajectory diagnosed in cGENIE for each RCP/ECP pathway.

Our simulations of carbon dioxide capture are designed to represent two distinct CDR deployment modes. In simulations of CDR via enhanced rock weathering (ERW), we specify an initial capture rate (in $GtCO_2$ y^{-1}) which is then translated into a removal of CO_2 from the atmosphere and a corresponding flux of dissolved inorganic carbon (DIC) and alkalinity to the coastal ocean. We simulate ERW using two distinct feedstocks, with idealized stoichiometries for natural silicate and carbonate minerals:

$$CaSiO_3 + 2CO_2 + 3H_2O \rightarrow Ca^{2+} + 2HCO_3^- + H_4SiO_4 \ , \qquad \text{[Eq. S6]}$$

$$CaCO_3 + CO_2 + H_2O \rightarrow Ca^{2+} + 2HCO_3^- \ . \qquad \text{[Eq. S7]}$$

Fluxes of DIC and alkalinity to the ocean per ton of CO_2 removed are controlled according to the stoichiometries shown, and are routed to the ocean according to simulated topological network (STN) data [8]. Note that on arbitrarily long timescales the formation and burial of carbonate minerals in marine sediments (e.g., the reverse of carbonate dissolution) releases CO_2, undoing most or all of the capture associated with ERW using carbonate rock and around half of the capture associated with ERW using silicate feedstock.

We also simulate an alternative carbon cycle intervention meant to represent direct CDR or mitigation of emissions in excess of that implied by a given RCP pathway, in which we reduce CO_2 emission rates globally by a specified value relative to the control emission rates for a given RCP. The key difference between this idealized style of CDR deployment and those associated with ERW is that it is assumed that the carbon is instantaneously and permanently removed from the surface system rather than being transiently repartitioned into a non-radiative but potentially labile form of surface carbon (some of which will ultimately be removed from Earth's surface). This style of intervention could be viewed as consistent with a variety of distinct strategies but is most directly analogized to direct air capture and storage (DACS) or emissions mitigation. We explore a wide range of CDR deployment scales between 0.5 – 40 $GtCO_2$ y^{-1}, a range meant to be inclusive of relatively modest CDR deployment on the low end to a deployment scale sufficient to offset most or all of global anthropogenic CO_2 emissions on the other.

Theoretical formulation of carbon leakage. Carbon capture and leakage through NETs can be defined based on fluxes between the major carbon reservoirs at Earth's surface relative to a deployed carbon cycle intervention, represented here as J_{CDR} but meant to incapsulate both direct carbon dioxide removal and mitigated fossil fuel emissions. Below we develop a basic framework for CO_2 capture efficiency as well as carbon leakage resulting from anthropogenic carbon cycle intervention.

The total mass of carbon that is transferrable on anthropogenic timescales, C_{all} (mol), can be defined as the summed mass of carbon in three reservoirs:≈

$$C_{all} = C_{atm} + C_{ocn} + C_{land} \ , \qquad \text{[Eq. S8]}$$

where C_{atm}, C_{ocn} and C_{land} represent the carbon reservoir size (mol) of the atmosphere, ocean, and land, respectively. Time-integrated flows of carbon caused by CDR deployment (or mitigated emissions) can be defined based on the difference in reservoir size between a given intervention experiment (*exp*) and a corresponding control simulation (*ctrl*):

$$\Delta C_X = C_X^{exp} - C_X^{ctrl} \ , \qquad \text{[Eq. S9]}$$

where X = *all*, *atm*, *ocn* or *land*. Given that carbon mass balance must be maintained with or without CDR/mitigated emissions:

$$\Delta C_{all} = \Delta C_{atm} + \Delta C_{ocn} + \Delta C_{land} \ . \qquad \text{[Eq. S10]}$$

We can then define capture efficiency with reference to the long-term impact on the atmospheric carbon reservoir as:

$$\eta_{CDR} = \frac{-\Delta C_{atm}}{\int_{2030}^{t} J_{CDR} dt'} \ , \qquad \text{[Eq. S11]}$$

We can also explicitly link the cumulative impact of CDR or mitigated emissions on the mass of atmospheric carbon with a corresponding alkalinity change in the ocean (ΔA_{ocn}, mol) associated with direct removal or mitigated emissions (base) and CDR through enhanced silicate weathering (ESW) or enhanced carbonate weathering (ECW).

$$\Delta A_{ocn} = \begin{cases} 0 & (\text{CDR = base}) \\ \int_{2030}^{t} J_{CDR} dt' & (\text{CDR = ESW}) \\ 2\int_{2030}^{t} J_{CDR} dt' & (\text{CDR = ECW}) \end{cases} \ , \qquad \text{[Eq. S12]}$$

We can also relate the time-integrated change in total Earth surface carbon mass to cumulative CO_2 removal or mitigated emissions across the same range of intervention styles:

$$\Delta C_{all} = \begin{cases} -\int_{2030}^{t} J_{CDR} dt' & (\text{CDR = base}) \\ 0 & (\text{CDR = ESW}) \\ \int_{2030}^{t} J_{CDR} dt' & (\text{CDR = ECW}) \end{cases} \ . \qquad \text{[Eq. S13]}$$

Note that Eq. S13 assumes timescales less than 10^5 years. Meanwhile, the land and ocean carbon reservoirs do not exchange directly but only through the atmosphere reservoir. Thus:

$$\Delta C_{land} = -\int_{2030}^{t} (J_{land\text{-}air}^{exp} - J_{land\text{-}air}^{ctrl}) dt' \ , \qquad \text{[Eq. S14]}$$

$$\Delta C_{ocn} = \Delta A_{ocn} - \int_{2030}^{t} (J_{sea\text{-}air}^{exp} - J_{sea\text{-}air}^{ctrl}) dt' \ . \qquad \text{[Eq. S15]}$$

Using Eqs. S11-S15, we can express capture efficiency with respect to carbon fluxes:

$$\eta_{CDR} = \frac{\int_{2030}^{t} \left[J_{CDR} - (J_{sea\text{-}air}^{exp} - J_{sea\text{-}air}^{ctrl}) - (J_{land\text{-}air}^{exp} - J_{land\text{-}air}^{ctrl}) \right] dt'}{\int_{2030}^{t} J_{CDR} dt'} \ . \qquad \text{[Eq. S16]}$$

Carbon leakage can then be defined as:

$$p_{CDR} \equiv 1 - \eta_{CDR} = \frac{\int_{2030}^{t} \left[(J_{sea\text{-}air}^{exp} - J_{sea\text{-}air}^{ctrl}) + (J_{land\text{-}air}^{exp} - J_{land\text{-}air}^{ctrl}) \right] dt'}{\int_{2030}^{t} J_{CDR} dt'} \ , \qquad \text{[Eq. S17]}$$

We define a baseline "Earth system leakage" (CDR = base in the expressions above) which does not involve any direct impacts on ocean chemistry. Then additional carbon leakage during ERW, Δp_{ERW}, can be defined using Eqs. S16-S17:

$$\Delta p_{ERW} = \begin{cases} p_{ESW} - p_{base} = 1 - \frac{\Delta' C_{ocn}}{\Delta' A_{ocn}}\left(1 + \frac{\Delta' C_{land}}{\Delta' C_{ocn}}\right) & \text{(ERW = ESW)} \\ p_{ECW} - p_{base} = 2\left[1 - \frac{\Delta' C_{ocn}}{\Delta' A_{ocn}}\left(1 + \frac{\Delta' C_{land}}{\Delta' C_{ocn}}\right)\right] & \text{(ERW = ECW)} \end{cases}, \quad \text{[Eq. S18]}$$

where Δ' indicates the difference from a corresponding baseline (direct removal or mitigated emissions) scenario:

$$\Delta' C_X = C_X^{ERW} - C_X^{base} \; . \quad \text{[Eq. S19]}$$

Here, ERW can denote either ESW or ECW. Our ensemble of simulations with stochastic slab biosphere parameterization suggests $\Delta' C_{land}/\Delta' C_{ocn} < 0.05$ and thus we can assume $\Delta' C_{ocn} >> \Delta' C_{land}$. Therefore, regardless of the response of the terrestrial biosphere we can simplify the expression for residual carbon leakage during ERW to:

$$\Delta p_{ERW} = \begin{cases} 1 - \frac{\Delta' C_{ocn}}{\Delta' A_{ocn}} & \text{(ERW = ESW)} \\ 2\left[1 - \frac{\Delta' C_{ocn}}{\Delta' A_{ocn}}\right] & \text{(ERW = ECW)} \end{cases}, \quad \text{[Eq. S20]}$$

Using the time-integrated global carbon flux and reservoir shifts from our ensemble of simulations and Eq. (S20) results in:

$$\frac{\Delta' C_{ocn}}{\Delta' A_{ocn}} \approx 0.9 \; , \quad \text{[Eq. S21]}$$

$$\Delta p_{ECW} \approx 2\Delta p_{ESW} \; , \quad \text{[Eq. S22]}$$

This is consistent with the near-term equilibrium values for residual carbon leakage given in Main Text Fig. 2. In both cases the carbon storage efficiency is greater than that conventionally assumed [9, 10], which we suggest results from transport of carbon into the ocean interior.

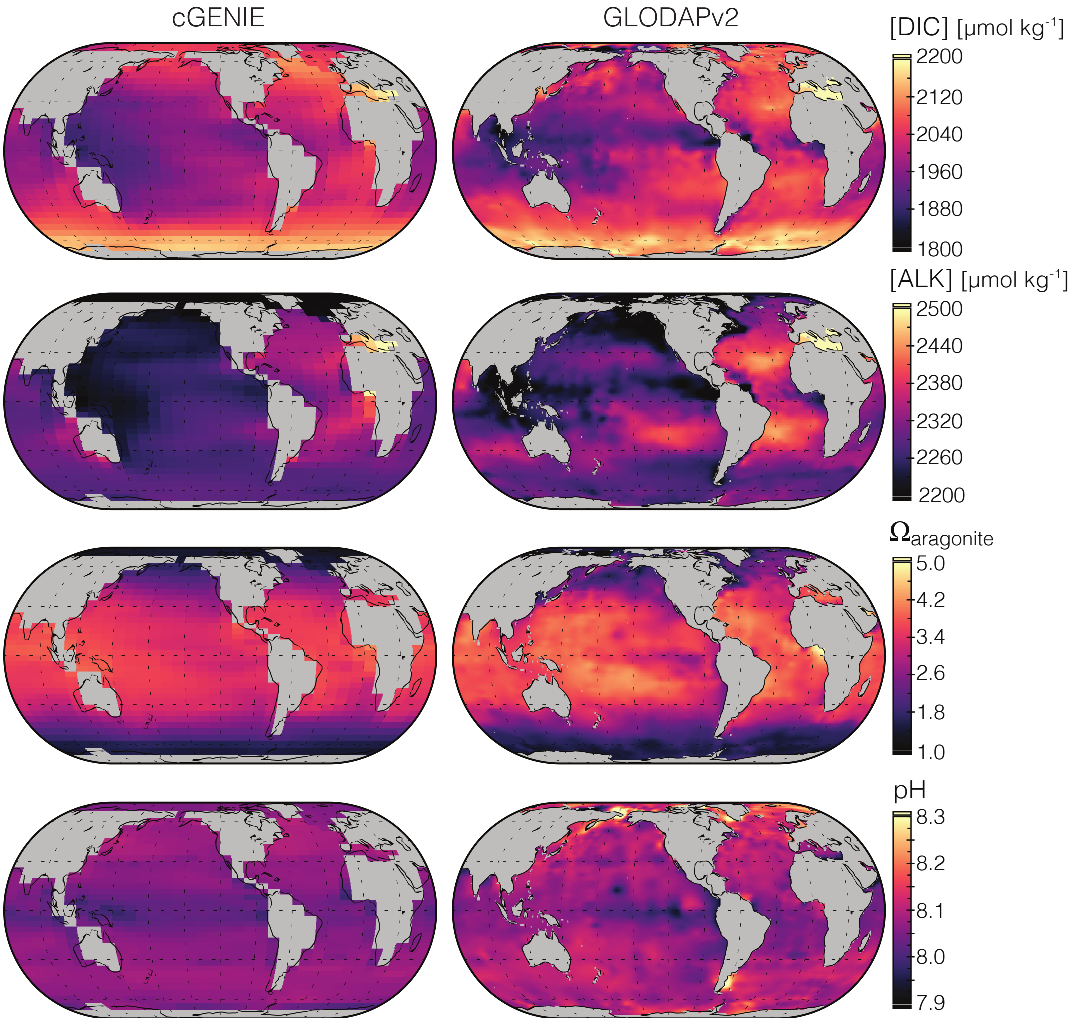

Figure S1. Surface ocean carbonate chemistry parameters in historical cGENIE simulation compared to gridded observational data. Shown at left are year 2000 results for concentrations of dissolved inorganic carbon ([DIC]), alkalinity ([ALK]), aragonite saturation state ($\Omega_{aragonite}$), and pH. Shown on the right are observational data from the GLODAPv2 dataset [11].

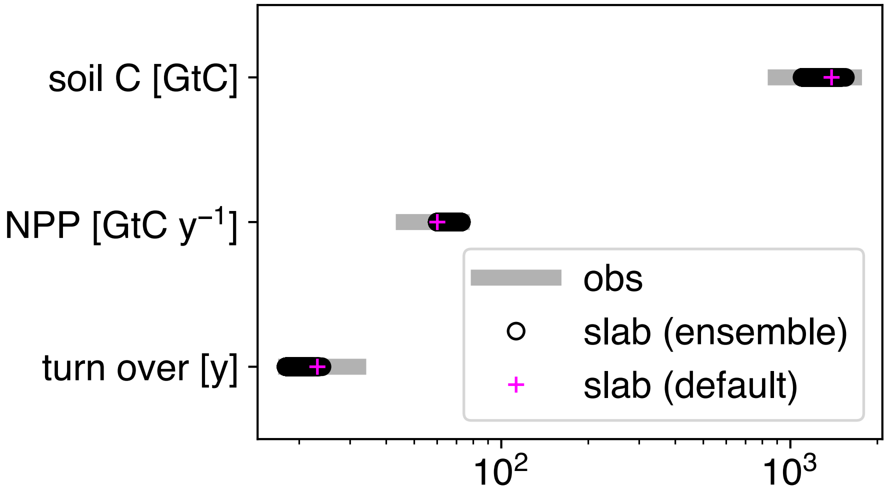

Figure S2. Comparison of Earth system model results with modern empirical observations of soil organic carbon (soil C), aboveground net primary productivity (NPP), and vegetation turnover time. Bars show modern observations [12], while crosses and open circles show Earth system model results using default slab biosphere parameters and the range for our filtered ensemble, respectively.

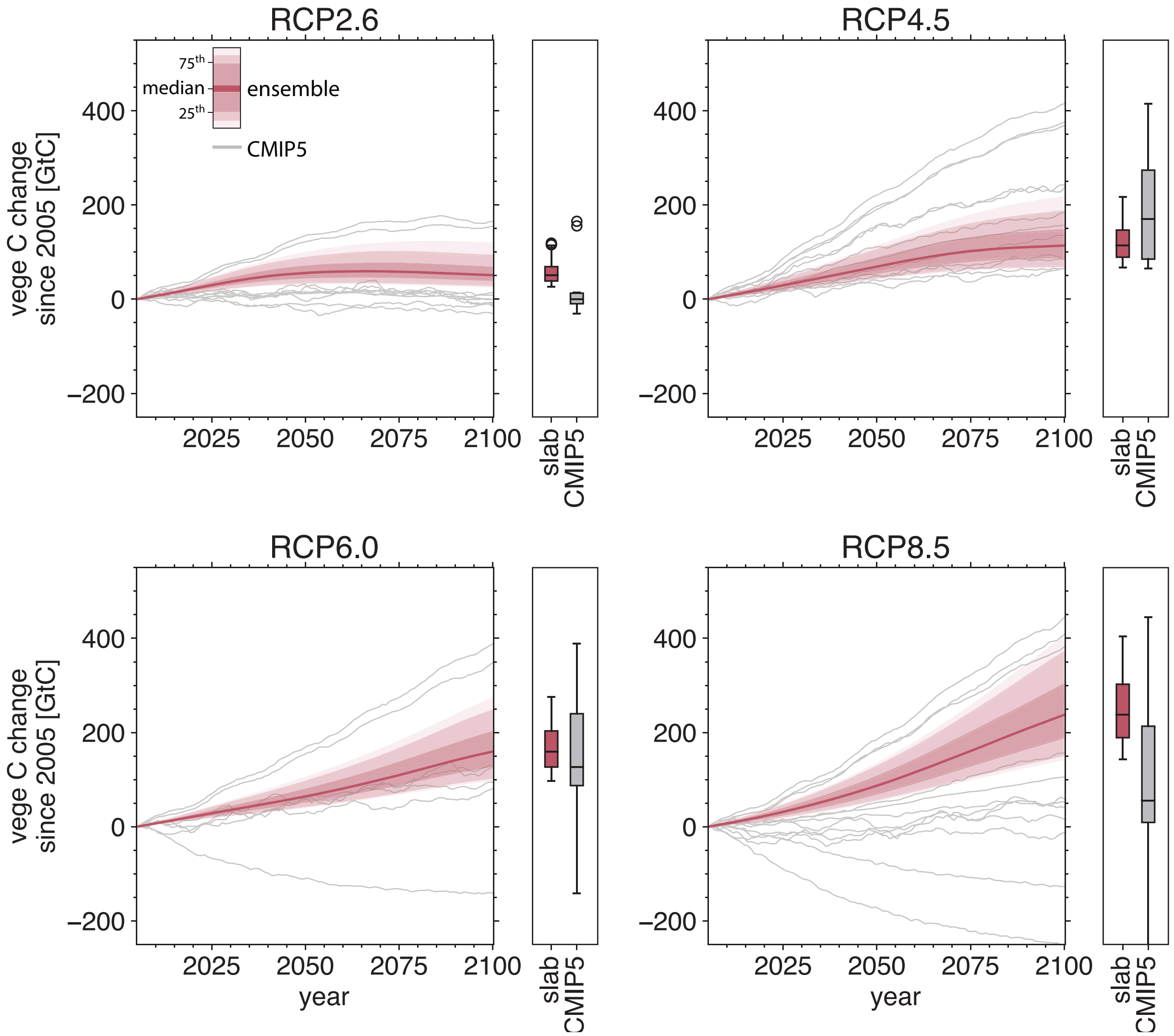

Figure S3. Comparison of Earth system model results with CMIP5 model range for aboveground vegetation carbon through the next century. Shaded envelope shows large-ensemble results from our intermediate complexity Earth system model (*n* = 980), while solid lines show individual members of the CMIP5 ensemble (https://esgf-node.llnl.gov/search/cmip5/).

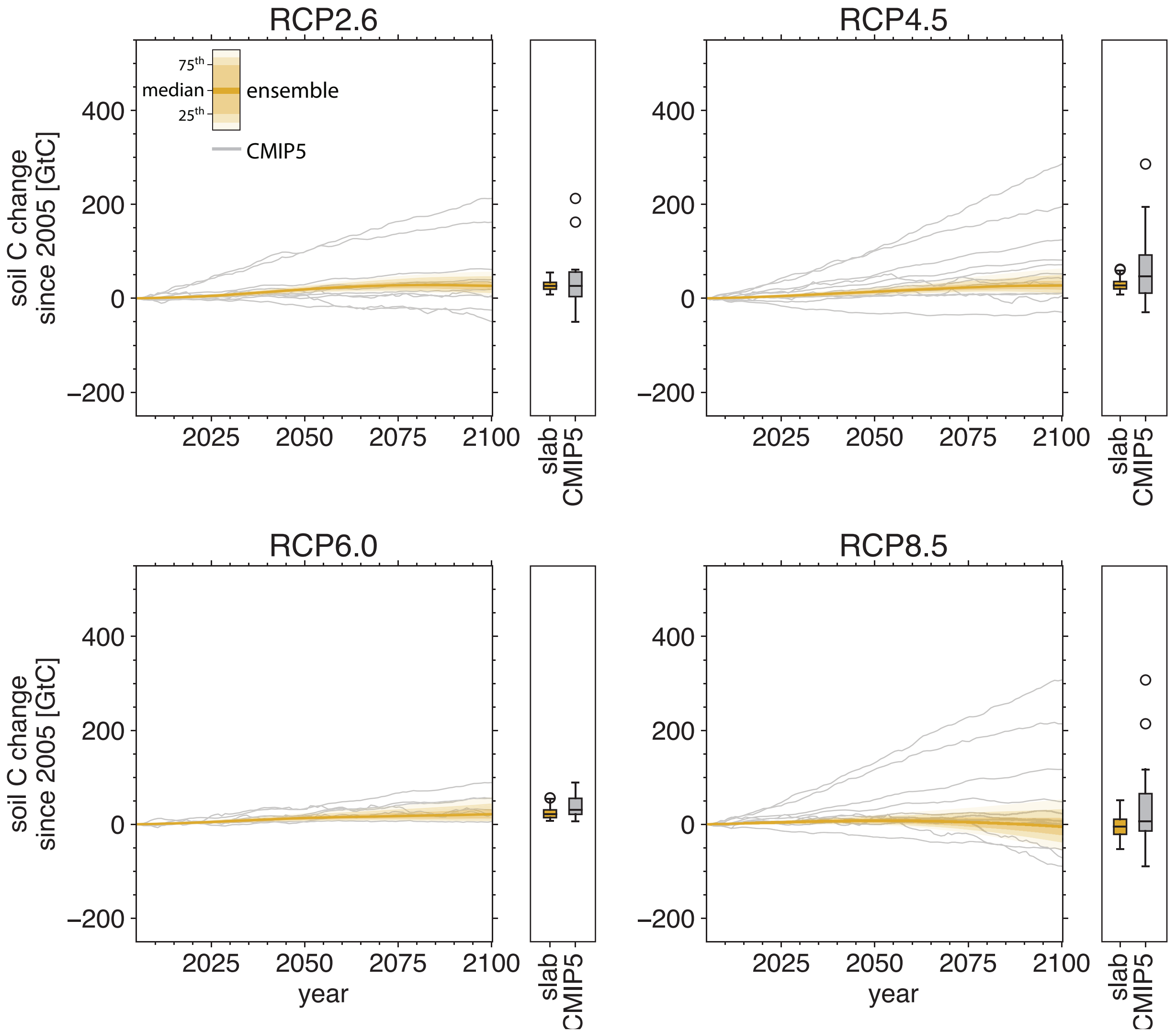

Figure S4. Comparison of Earth system model results with CMIP5 model range for soil organic carbon through the next century. Shaded envelope shows large-ensemble results from our intermediate complexity Earth system model ($n = 980$), while solid lines show individual members of the CMIP5 ensemble (https://esgf-node.llnl.gov/search/cmip5/).

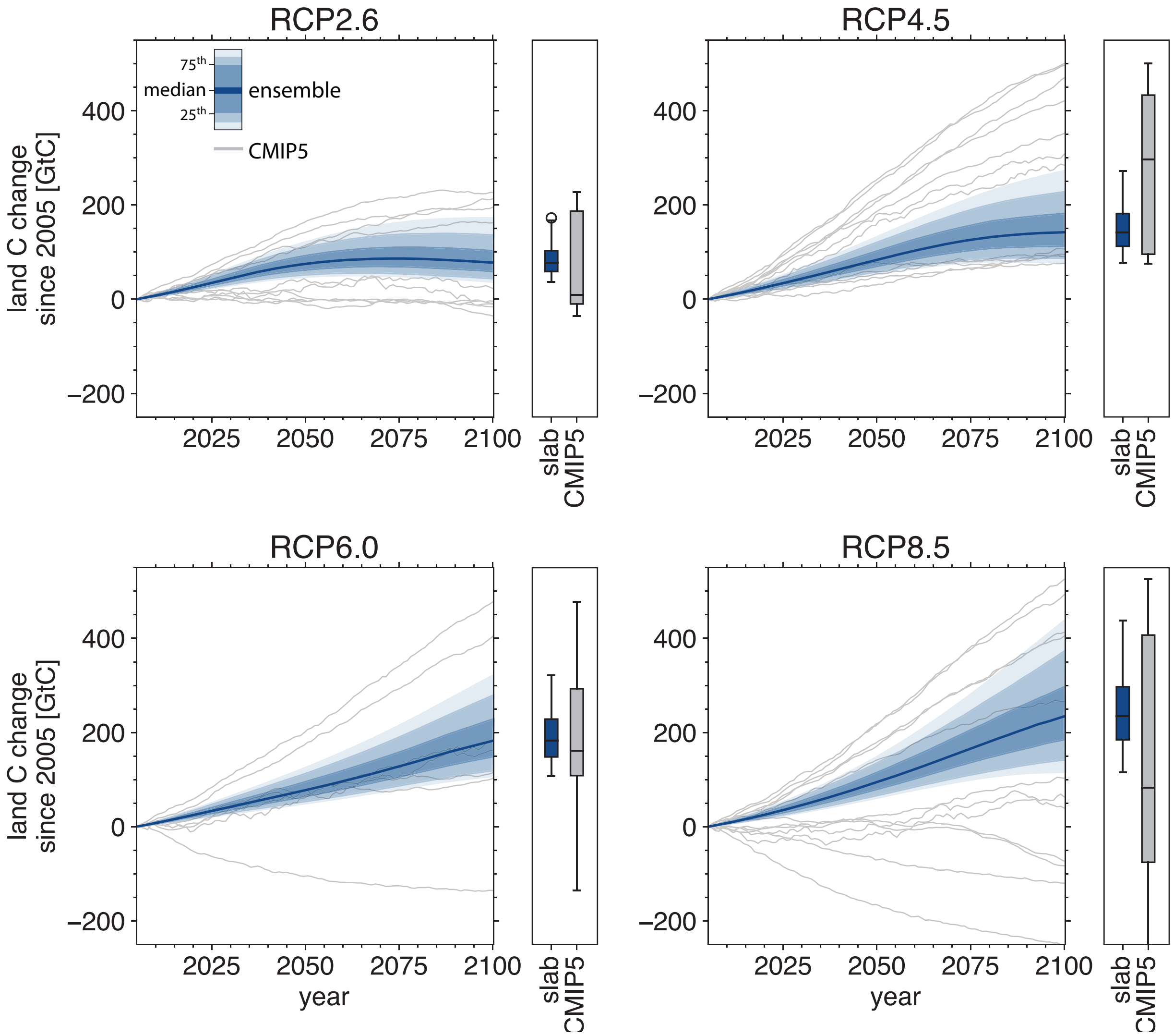

Figure S5. Comparison of Earth system model results with CMIP5 model range for total land surface carbon through the next century. Shaded envelope shows large-ensemble results from our intermediate complexity Earth system model ($n = 980$), while solid lines show individual members of the CMIP5 ensemble (https://esgf-node.llnl.gov/search/cmip5/).

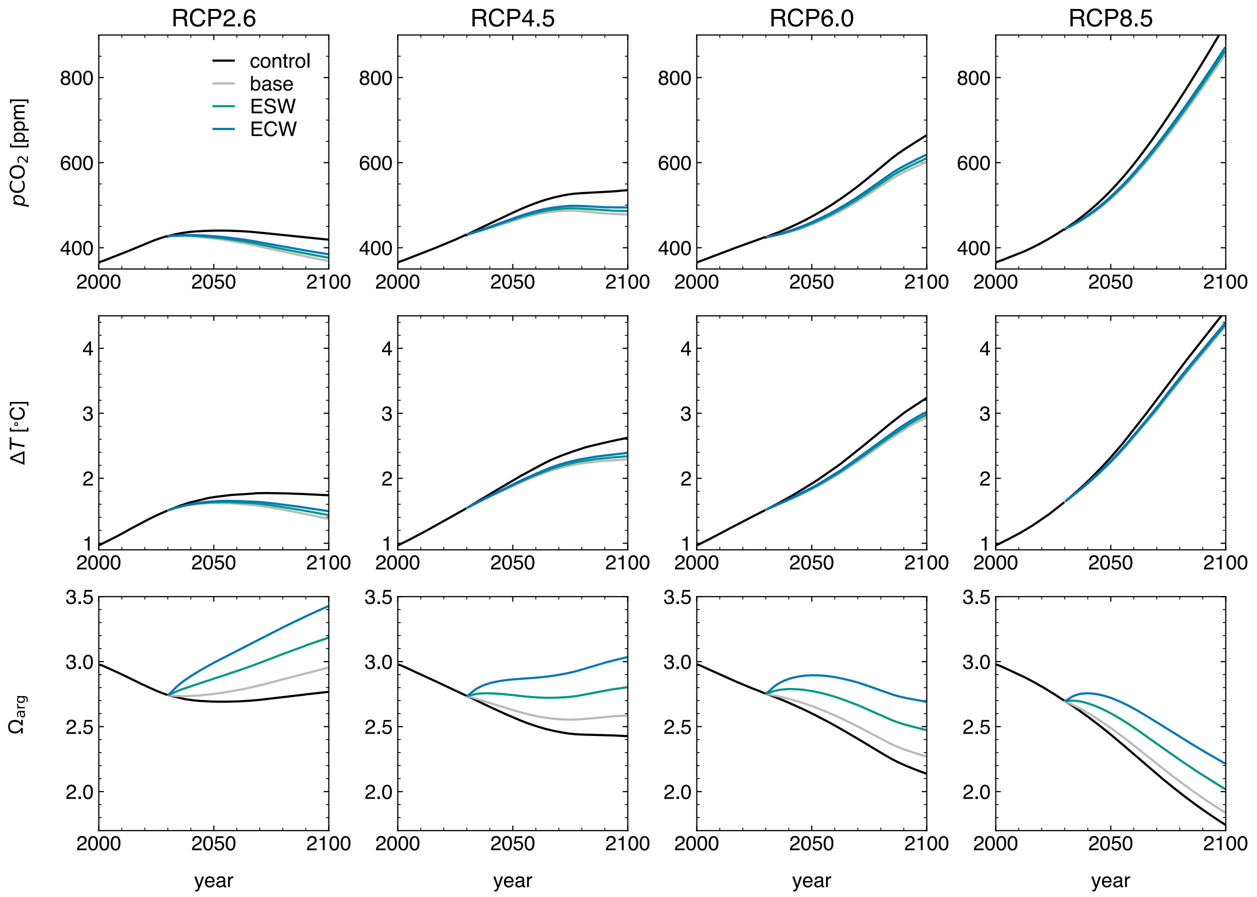

Figure S6. Atmospheric carbon dioxide (pCO_2), global average warming since the preindustrial period (ΔT), and average surface ocean aragonite saturation state (Ω_{arg}) for a range of emission and CDR scenarios. Results are shown for four Representative Concentration Pathway (RCP) scenarios (left-right), and for control runs with no CDR (black), direct air capture and storage and/or additional mitigated emissions (grey), enhanced silicate weathering (ESW; green), and enhanced carbonate weathering (ECW; blue). All CDR scenarios are for a 10 $GtCO_2$ y^{-1} deployment beginning in 2030. Values for ΔT are calculated relative to the period 1850-1900.

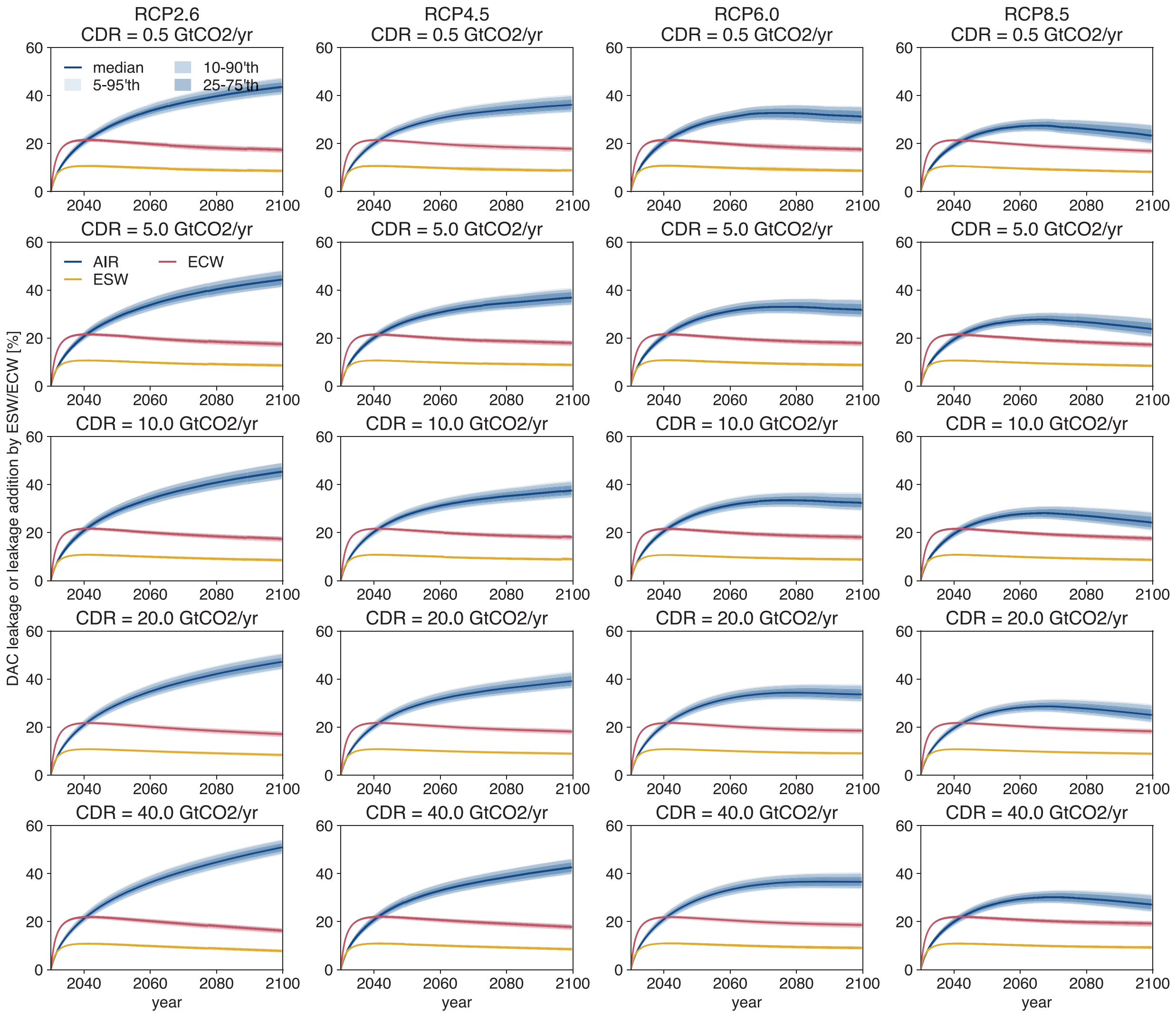

Figure S7. Carbon leakage through 2100 across a range of emission and carbon dioxide removal (CDR) scenarios, shown as a time-integrated percentage relative to CDR deployment level. Black curves and error envelopes show the ensemble median and uncertainty on baseline (modulated emissions) response, while red and yellow curves and error envelopes show ensemble median and uncertainty for the residual enhanced carbonate weathering (ECW) and enhanced silicate weathering (ESW), respectively, after correcting to the baseline response.

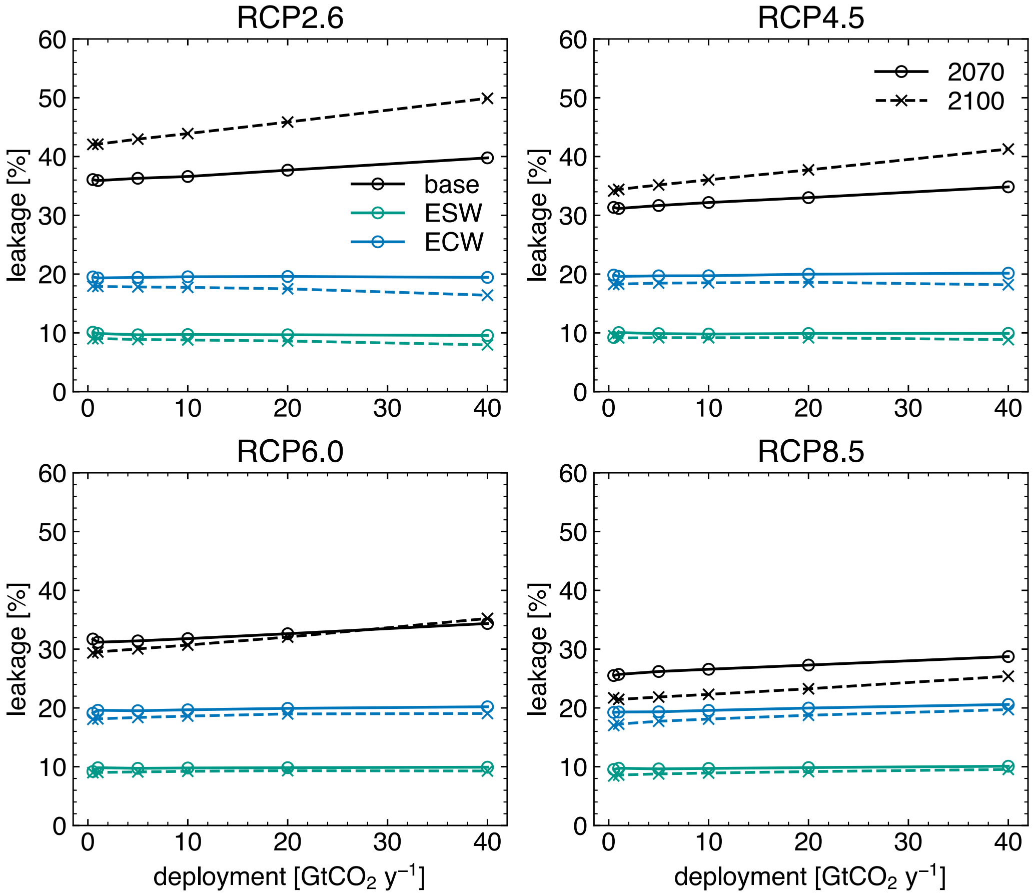

Figure S8. Carbon leakage as a function of deployment level of carbon dioxide removal (CDR) or additional mitigated emissions. Results are shown for each Representative Concentration Pathway (RCP) scenario in model years 2070 and 2100, for baseline (base) intervention, enhanced silicate weathering (ESW) and enhanced carbonate weathering (ECW).

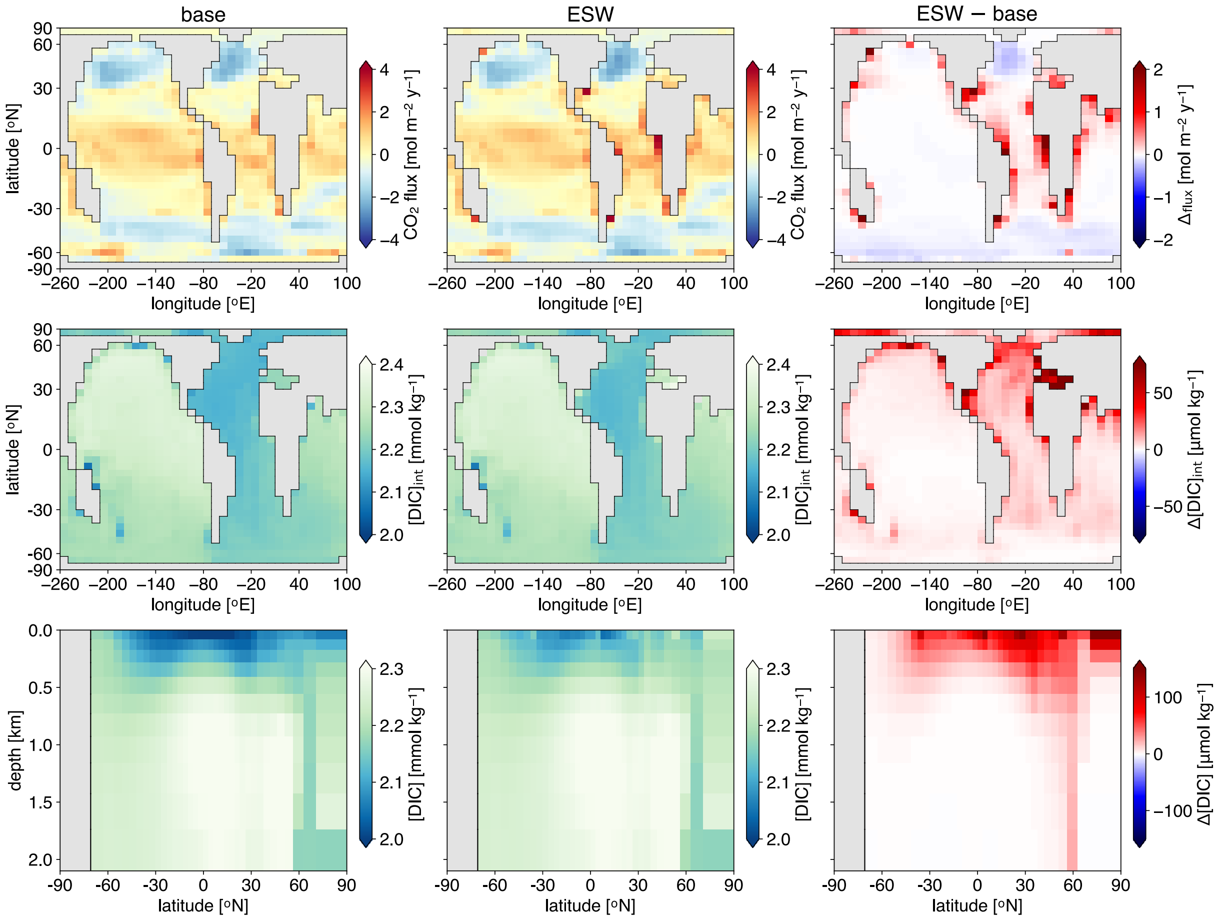

Figure S9. Ocean carbon cycle response to carbon dioxide removal in our Earth system model for RCP2.6. Shown at left are sea-air CO_2 fluxes (top), depth-integrated inventory of dissolved inorganic carbon ($[DIC]_{int}$; middle), and zonally averaged DIC concentrations (bottom) for the baseline (modulated emissions) case and the enhanced silicate weathering (ESW) scenario. Shown at right are anomaly plots of sea-air flux (top), depth-integrated DIC inventory (middle), and zonally averaged DIC (bottom) between the ESW scenario and the equivalent modulated emissions case. Results are shown for year 2070 and a continuous CDR deployment level of 10 $GtCO_2$ y^{-1} starting in 2030. Note that DIC concentration/anomaly results (D-I) are shown excluding the uppermost grid cell (80m).

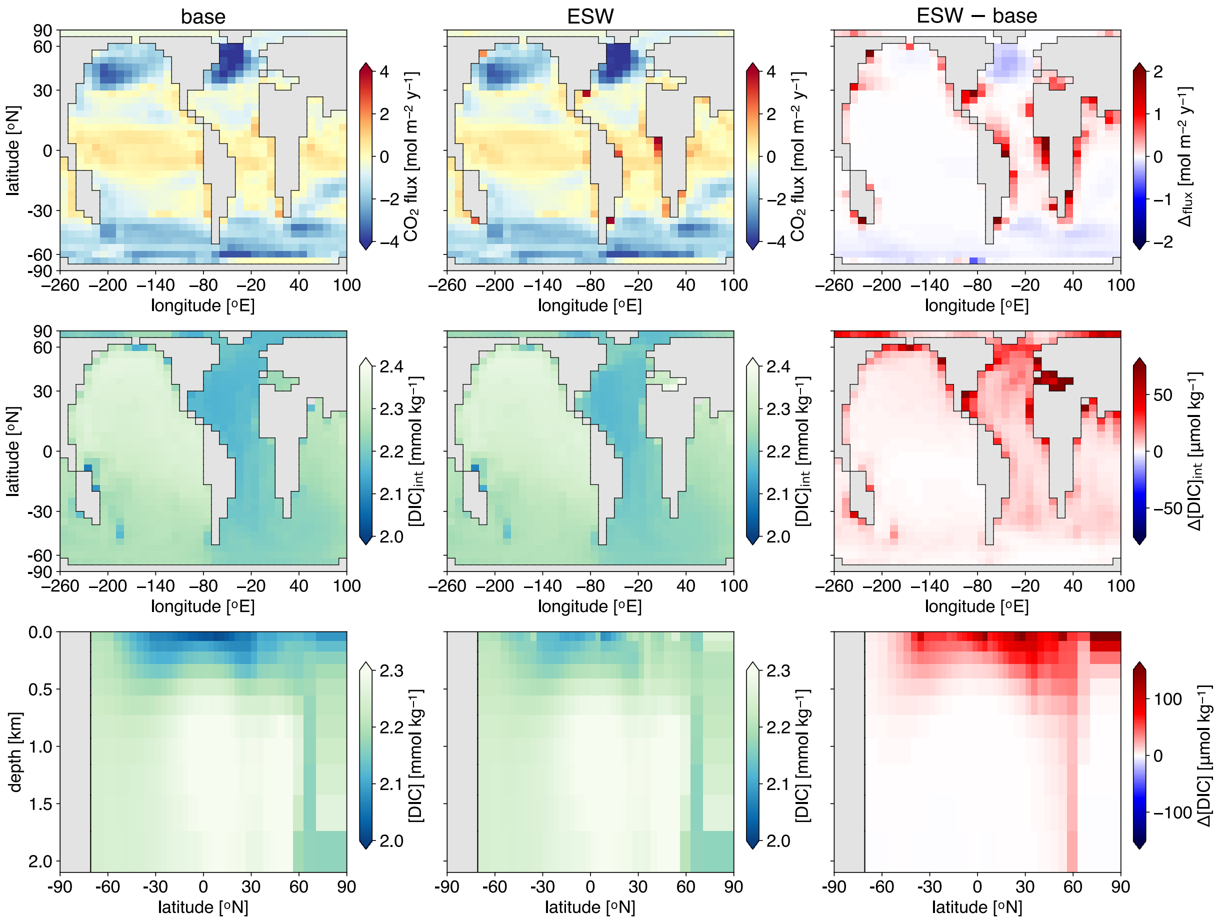

Figure S10. Ocean carbon cycle response to carbon dioxide removal in our Earth system model for RCP6.0. Shown at left are sea-air CO_2 fluxes (top), depth-integrated inventory of dissolved inorganic carbon ($[DIC]_{int}$; middle), and zonally averaged DIC concentrations (bottom) for the baseline (modulated emissions) case and the enhanced silicate weathering (ESW) scenario. Shown at right are anomaly plots of sea-air flux (top), depth-integrated DIC inventory (middle), and zonally averaged DIC (bottom) between the ESW scenario and the equivalent modulated emissions case. Results are shown for year 2070 and a continuous CDR deployment level of 10 $GtCO_2$ y^{-1} starting in 2030. Note that DIC concentration/anomaly results (D-I) are shown excluding the uppermost grid cell (80m).

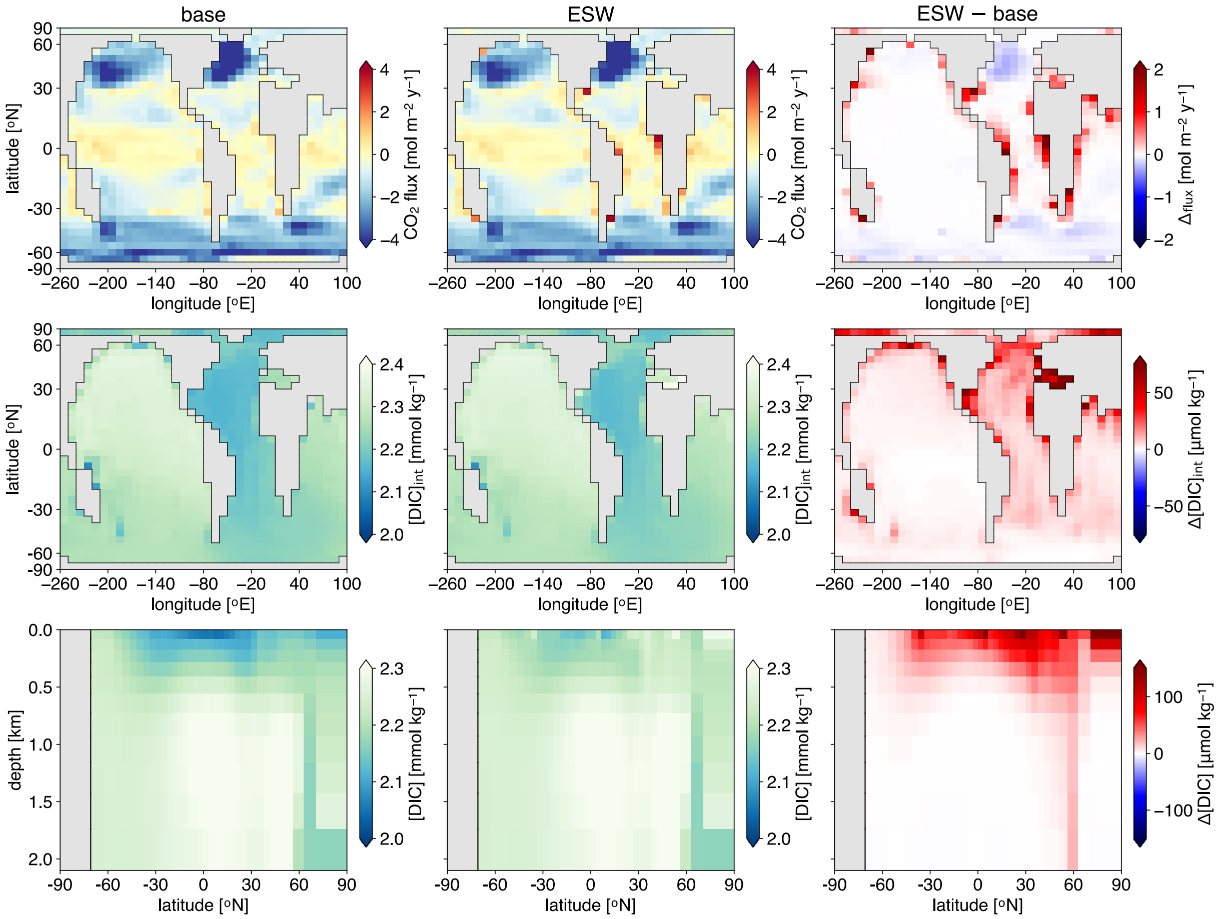

Figure S11. Ocean carbon cycle response to carbon dioxide removal in our Earth system model for RCP8.5. Shown at left are sea-air CO_2 fluxes (top), depth-integrated inventory of dissolved inorganic carbon ($[DIC]_{int}$; middle), and zonally averaged DIC concentrations (bottom) for the baseline (modulated emissions) case and the enhanced silicate weathering (ESW) scenario. Shown at right are anomaly plots of sea-air flux (top), depth-integrated DIC inventory (middle), and zonally averaged DIC (bottom) between the ESW scenario and the equivalent modulated emissions case. Results are shown for year 2070 and a continuous CDR deployment level of 10 $GtCO_2$ y^{-1} starting in 2030. Note that DIC concentration/anomaly results (D-I) are shown excluding the uppermost grid cell (80m).

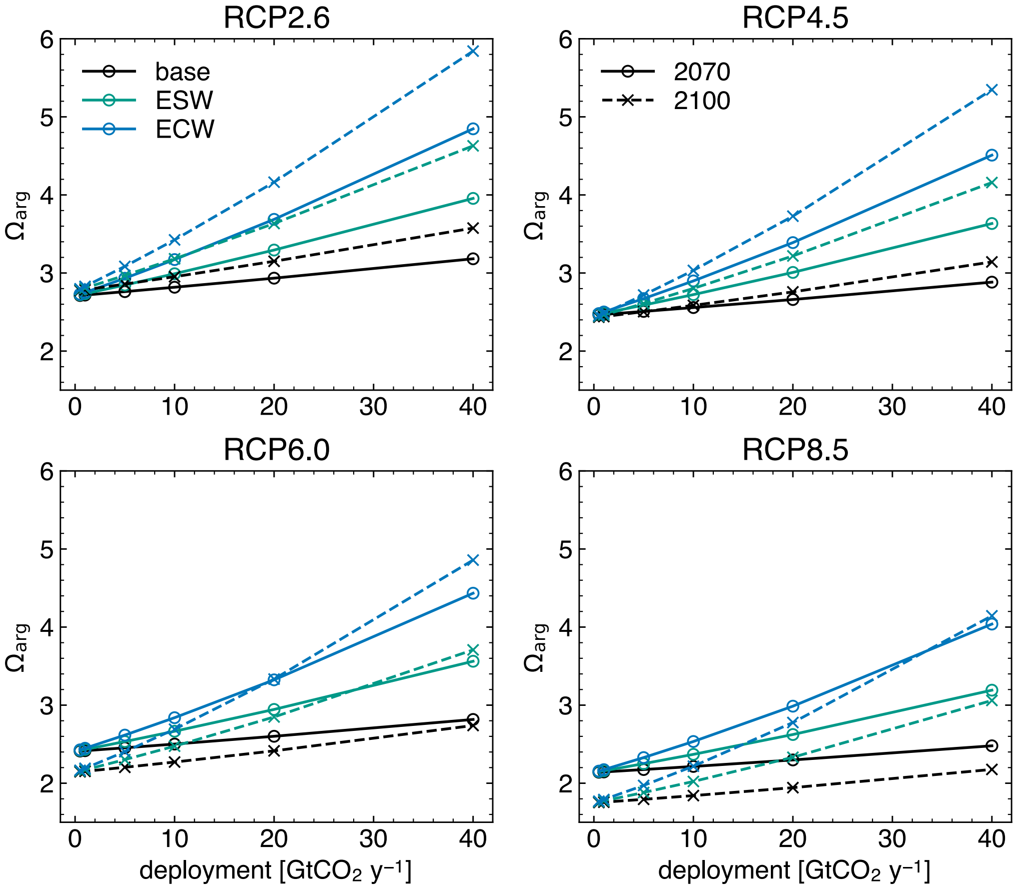

Figure S12. Global average surface ocean aragonite saturation state (Ω_{arg}) as a function of deployment level of carbon dioxide removal (CDR) or additional mitigated emissions. Results are shown for each Representative Concentration Pathway (RCP) scenario in model years 2070 and 2100, for baseline (base) intervention, enhanced silicate weathering (ESW) and enhanced carbonate weathering (ECW).

Table S1. Governing equations for the terrestrial-biosphere box module.

Parameter (symbol [units])	Equation
Vegetation (V [GtC])	$\frac{dV(t)}{dt} = N(t) - L(t)$
Soil carbon (S [GtC])	$\frac{dS(t)}{dt} = L(t) - R(t)$
Net primary production (N [GtC y^{-1}]) [a]	$N(t) = N_0 \left[1 + \beta \ln \left\{ \frac{pCO_2(t)}{pCO_2^{ref}} \right\} \right]$
Litterfall (L [GtC y^{-1}])	$L(t) = \frac{V(t)}{\Lambda}$
Respiration (R [GtC y^{-1}]) [b]	$R(t) = \Gamma S(t) Q_{10}^{\frac{T(t)-T_0}{10}}$
Turn over (Λ [y])	$\Lambda = aV(t) + b$

[a] pCO_2 is in units of ppm and pCO_2^{ref} is a reference pCO_2 (365 ppm).

[b] T is the global average land surface temperature excluding Antarctica and Greenland (°C) and T_0 is a reference T (15 °C).

Table S2. Parameter ranges examined and values chosen for the terrestrial-biosphere box module (Table S1).

Parameter [units]	Examined plausible range [a]	Default value [b]
N_0 [GtC y^{-1}]	60 – 80	60
β [dimensionless]	0.3 – 0.9	0.42
Γ [y^{-1}]	0.04 – 0.07	0.04
Q_{10} [dimensionless]	1.0 – 5.0	2.6
a [y GtC^{-1}]	−0.02 – 0.004	−0.0056
b [y]	5 – 18	18

[a] Ranges cover values reported in [13].

[b] The combination of the parameter values listed in the third column is adopted as default, which yields estimates consistent with observations and CMIP5 forecasts.

SI References

1. Marsh, R., et al., *Incorporation of the C-GOLDSTEIN efficient climate model into the GENIE framework: "eb_go_gs" configurations of GENIE.* Geoscientific Model Development, 2011. **4**: p. 957-992.
2. Edwards, N.R. and R. Marsh, *Uncertainties due to transport-parameter sensitivity in an efficient 3-D ocean-climate model.* Climate Dynamics, 2005. **24**: p. 415-433.
3. Ridgwell, A. and J.C. Hargreaves, *Regulation of atmospheric CO2 by deep-sea sediments in an Earth system model.* Global Biogeochemical Cycles, 2007. **21**(2).
4. Ridgwell, A. and D.N. Schmidt, *Past constraints on the vulnerability of marine calcifiers to massive carbon dioxide release.* Nature Geoscience, 2010. **3**: p. 196-200.
5. Reinhard, C.T., et al., *Oceanic and atmospheric methaen cycling in the cGENIE Earth system model.* Geoscientific Model Development, 2020.
6. Ridgwell, A., et al., *Marine geochemical data assimilation in an efficient Earth System Model of global biogeochemical cycling.* Biogeosciences, 2007. **4**(1): p. 87-104.
7. Meinshausen, M., et al., *The RCP greenhouse gas concentrations and their extensions from 1765 to 2300.* Climatic Change, 2011. **109**: p. 213-241.
8. Colbourn, G., A. Ridgwell, and T.M. Lenton, *The Rock Geochemical Model (RokGeM) v0.9.* Geoscientific Model Development, 2013. **6**: p. 1543-1573.
9. Renforth, P., *The negative emission potential of alkaline materials.* Nature Communications, 2019. **10**.
10. Renforth, P. and G. Henderson, *Assessing ocean alkalinity for carbon sequestration.* Reviews of Geophysics, 2017. **55**: p. 636-674.
11. Olsen, A., et al., *The Global Ocean Data Analysis Project version 2 (GLODAPv2) - An internall consistent data product for the world ocean.* Earth System Science Data, 2019. **8**: p. 297-323.
12. Todd-Brown, K.E.O., et al., *Causes of variation in soil carbon simulations from CMIP5 Earth system models and comparison with observations.* Biogeosciences, 2013. **10**: p. 1717-1736.
13. Arora, V.K. and H.D. Matthews, *Characterizing uncertainty in modeling primary terrestrial ecosystem processes.* Global Biogeochemical Cycles, 2009. **23**.
